# Supplementary material for: Assessing the influence of the modifiable areal unit problem on Bayesian disease mapping in Queensland, Australia
Source: PLoS One. 2025 Jan 28;20(1):e0313079. doi: 10.1371/journal.pone.0313079 (PMC11774366; doi:10.1371/journal.pone.0313079)
Supplement: Attachment — Submitted filename: Response to Reviewers comments.pdf [file pone.0313079.s003.pdf]

Response to reviewer's comments

Thanks for the comments. I have addressed each comment and made some modifications to the article as shown within each response. The list of references used in addressing the comments are added at the end of all the responses. The list at the end of this document is the complete list of references in the revised manuscript.

Reviewer #2: 1. Why is it significant to investigate the impact of the MAUP in regions with sparse data, particularly in countries like Australia with less populated areas?

Response:

Disease mapping is a popular tool for showing the patterns of disease in small areas of a country as it can inform people and policy makers about the patterns of certain disease incidence and survival in the regions they live. This identification of at-risk areas can then aid in improving disease survival (e.g. through targeted early detection) or reduce disease incidence (e.g. through targeted prevention measures). It is important to understand differences by small populations (in Australia, this is often of the order of 10,000 people) to avoid missing important differences between subpopulations. Sparsely populated areas often have poorer access to health facilities and poorer health outcomes, and so it is particularly important to have an accurate understanding of the variation in these areas.

However, if the way data are aggregated can drastically change statistical results when data are sparse, then using different spatial units can lead to different interpretations of the same data, affecting policy decisions and resource allocation. Accurate small area estimates are crucial in sparsely populated regions to understand localized phenomena. MAUP can distort these estimates, leading to misleading conclusions about trends and patterns. Hence it is significant to investigate the impact of MAUP when data are sparse, so that the appropriate level of data aggregation can be used for disease mapping, which will then lead to accurate estimates to facilitate decision making for health managers, policy makers and resource allocation.

This information is briefly added to the Introduction section of the manuscript (highlighted):

Internationally, there has been some examination of MAUP for less common diseases. In the United States MAUP was evaluated when modelling breast cancer incidence in Indiana State Cancer Registry from 2010 to 2015, but even at the smallest levels examined, areas had between 600 to 3000 residents [16]. Their suggestion to avoid MAUP was to disaggregate counts to approximate individual-point locations, as occurred for a New Hampshire study on birth defects [17]. This used Restricted and Controlled Monte Carlo (RCMC) to disaggregate area-level counts by randomly placing individuals in pixels within the original area, with a higher probability of being placed where the population was higher. Disadvantages included running multiple times to account for the inherent uncertainty in placement, and then being restricted to using analyses designed for individual data.

Investigating the impact of MAUP in regions with sparse data is crucial for accurate and reliable spatial analyses. In countries like Australia, where large areas have low population densities, this investigation is particularly important to inform policies and decisions that are equitable, effective, and tailored to the unique characteristics of these regions. Understanding MAUP ensures that data-driven decisions truly reflect the realities of these areas, leading to better outcomes for their inhabitants and environments.

Given the limited suggestions offered to date, there remains a significant opportunity to elucidate the influence of the MAUP within sparse data contexts. The impact of the MAUP has not previously been explored on outcomes with low counts and very sparse populations, such as cancer within rural and remote Australia. The present study aims to fill this research gap by exploring the implications of Bayesian spatial modeling applied to sparse data aggregated at diverse boundary levels across an entire state in Australia.

In this paper we explore the impact of the MAUP using Bayesian spatial models applied to synthetic-style cancer data, which has lower overall counts than previous studies, across different levels of aggregation in Queensland, Australia. We consider the impact on both spatial disease patterns and covariate inference.

2. How MAUP affects the analysis of spatially aggregated data? Why it is a concern in spatial analysis?

Response:

MAUP has been a well analysed phenomena, from the inception of spatial analysis using aggregated data, but it remains unsolved [9]. The Modifiable Areal Unit Problem (MAUP) affects the analysis of spatially aggregated data by introducing variability in statistical results based on how spatial units are defined and aggregated.

The term, MAUP, was used for the first time by Openshaw [2] showing the systematic variation in correlation values using different boundary systems for analysis. MAUP has since been examined in multiple studies. Since this is so-well established in the literature, we originally only briefly introduced MAUP in the Introduction, but we have now expanded to the following (highlighted):

The term, MAUP, was used for the first time by Openshaw and Taylor [2] showing the systematic variation in correlation values using different boundary systems for analysis. The MAUP may emerge from two different mechanisms [3]: the zoning effect, where the total number of areas is kept constant but boundaries are redefined; and the scaling effect, where the spatial resolution or aggregation level is altered leading to changes in the total number of areas. Analysing datasets from the same population using different zonations or scales may lead to inconsistent results, which is the essence of the MAUP [3]. Multiple cancer studies have demonstrated that different methods of aggregating health data led to varying interpretations of disease clusters (e.g. [4,5]), with different zoning schemes and scales of aggregation resulting in different clusters being identified and impacting conclusions about high-risk areas.

Some have argued that aggregation beyond the minimal level yields untrustworthy results [6] , while others believe there can be equally valid but different conclusions from analysing data at different aggregation levels [7]. Understanding the MAUP is crucial for analysis of spatial data at the small area level [8], since there are still several unresolved issues [9].

3. What advantages do area structures with moderate resolution offer in terms of dealing with the MAUP?

Response:

Moderate resolution provides a balanced level of detail, avoiding the extremes of overly fine or overly coarse aggregation. This balance helps to capture important spatial patterns without introducing excessive noise or losing significant information. This indirectly affects the MAUP, since it is determined by the estimates at different resolutions.

As mentioned in the Discussion and Conclusion of the article, the smallest aggregation level in Australia is not advantageous for modelling the disease pattern whereas going to higher aggregation levels can cause between-area spatial autocorrelation to vanish. We have further expanded on this point in the Discussion, as follows:

While modeling at the mesh block level can increase the sample size (i.e., the number of areas) and reduce heterogeneity within areas, it can also lead to situations where many areas have a small or zero number of cases or population, resulting in a loss of information and the potential for oversmoothing. This aligns with the findings in Fontanet et al. [16] where the smallest level failed to provide meaningful inference due to smaller populations and counts. Kok et al. [11] also found this to be the case when analysing hospitalisation rates for foot-related issues among the Indigenous population of Australia. Our findings, in conjunction with theirs, suggest that while aiming for the highest level of resolution may be optimal to reduce the biases of the MAUP, using mesh blocks can introduce other problems such as small numbers, zero populations, and computational inefficiencies. In small-area health studies, particularly those involving rare diseases or small populations, sparse data can lead to highly imprecise rate estimates. This imprecision is amplified by MAUP, causing substantial variability in the identification of disease clusters based on different spatial units [4,36]. As a result, health interventions and resource allocations might be misdirected due to the unreliability of the spatial analysis.

In addition, advantages of using the well-defined boundary levels of SA1 and SA2 also enables easy incorporation of additional demographic and socio-economic data into disease models [37]. Detailed spatial data at these levels can often be more effectively communicated to stakeholders, policymakers, and the public, facilitating better understanding and engagement.

With higher aggregations, we found that the spatial autocorrelation of the data is no longer significant (for SA3 and SA4 levels), so use of spatial modelling/smoothing may not be required. But since SA1 and SA2 level data has significant spatial autocorrelation, analysis of cancer incidence data at these levels should be performed using a model that accounts for spatial autocorrelation.

4. What types of Bayesian spatial models were employed in the study, and how do they account for spatial correlation and variability?

Response:

Bayesian hierarchical spatial models were employed in this study. These models are well suited for modelling disease data at the small area level, and we have now included full model details in the Statistical Methods section of the article, as follows:

Bayesian Spatial Model: BYM Model

The observed cancer counts from different small area aggregations are modelled using a very well-known Bayesian spatial modelling framework, a BYM model [15]. The BYM model is a type of generic three level Bayesian hierarchical model, where at the first stage the observed disease counts are modelled using an appropriate likelihood, the second stage models the spatial association by specifying appropriate structures and the third stage specifies the hyperprior distributions [1].

The BYM model incorporates extra variation at the second stage via two random effect terms: a) a spatially structured component and b) a spatially unstructured component. Inclusion of both spatially structured and unstructured random effects enable the disease rates to be smoothed at both a global and local level [28]. For the spatially structured term, the most commonly used approach for areal spatial data is the Gaussian Markov random field (GMRF) model which conditions area i within its neighbourhood [29,30], particularly in the form of the conditional autoregressive (CAR) prior. The detailed BYM model used is presented below:

Let Y_i be the observed lung cancer counts, which can be expressed under the general disease mapping framework using a Poisson likelihood [1]:

$$Y_i \sim \text{Poisson}\left(E_i e^{\mu_i}\right), \text{ for } i = 1, 2, \ldots, N \text{areas},$$

where E_i is the expected cancer counts in each area i. The present study will have different values of N for different boundary levels (Table 1). The log-relative risk, denoted μ_i, is often expressed as a regression equation:

$$\mu_i = \alpha + \boldsymbol{x}_i^T \boldsymbol{\beta} + \psi_i,$$

where the intercept α denotes an overall fixed effect, $\boldsymbol{\beta}$ is the covariate effects associated with the vector of covariates $\boldsymbol{x}_i$ relating to area i and ψ_i are the spatial random effects. In the context of this study, $\boldsymbol{x}_i$ represents the SEIFA IRSD quintiles for each area, where the first quintile (most disadvantaged) is considered as the reference category and $\boldsymbol{\beta}$ is hence a 4×1 vector representing the covariate effect of SEIFA IRSD quintiles 2, 3, 4, and 5. The Gaussian priors, $N(0, \sigma_\alpha^2)$ and $N(0, \sigma_\beta^2)$ are used for α and $\boldsymbol{\beta}$. The value of hyperparameters σ_α^2 and σ_β^2 are usually chosen to be a large number. In the present study, we have used the default priors and parameters provided by the `CARBayes` package ($\sigma_\alpha^2 = \sigma_\beta^2 = 1,000,000$) [31]. The random effect $\psi_i = u_i + v_i$, where u_i is a spatial random effect a CAR prior structure and v_i is the unstructured random effect. The conditional distribution of each u_i can be expressed as:

$$u_i | u_{j, i \neq j} \sim N\left(\frac{\sum_j w_{ij} u_j}{\sum_j w_{ij}}, \frac{\sigma_u^2}{\sum_j w_{ij}}\right),$$

where w_{ij} are the weights defining the relationship between area i and its neighbours such that:

$$w_{ij} = \begin{cases} 1, & \text{if areas } i \text{ and } j \text{ are adjacent} \\ 0, & \text{otherwise.} \end{cases}$$

The prior for the unstructured random component v_i is typically an independent normal distribution,

$$v_i \sim N(0, \sigma_v^2).$$

The hyperpriors placed on the variance terms σ_u^2 and σ_v^2 are Inverse-Gamma(a, b) with hyperparameters $a = 1$ (shape) and $b = 0.01$ (scale) [31].

5. What are the potential benefits of using SA1 or SA2 levels for spatial modelling according to the authors?

Response:

Disease data aggregated to defined boundary levels such as SA1 and SA2, which are well defined by the Australian Statistical Geography Standard (ASGS) provides several advantages for spatial modelling.

SA1 and SA2 offer a finer spatial resolution compared to larger geographic units allowing for more precise spatial analysis and identification of localised patterns and trends that might be missed with larger resolutions. Using the well-defined boundary levels such as SA1 and SA2 also makes it possible to access other socio-demographic and socio-economic data that can be useful in modelling the disease data and trying to find out influence of other covariates on the disease incidence [37]. Detailed spatial data at these levels can be more effectively communicated to stakeholders, policymakers, and the public, facilitating better understanding and engagement.

We have added an additional paragraph into the Discussion, as follows:

> While modeling at the mesh block level can increase the sample size (i.e., the number of areas) and reduce heterogeneity within areas, it can also lead to situations where many areas have a small or zero number of cases or population, resulting in a loss of information and the potential for oversmoothing. This aligns with the findings in Fontanet et al. [16] where the smallest level failed to provide meaningful inference due to smaller populations and counts. Kok et al. [11] also found this to be the case when analysing hospitalisation rates for foot-related issues among the Indigenous population of Australia. Our findings, in conjunction with theirs, suggest that while aiming for the highest level of resolution may be optimal to reduce the biases of the MAUP, using mesh blocks can introduce other problems such as small numbers, zero populations, and computational inefficiencies. In small-area health studies, particularly those involving rare diseases or small populations, sparse data can lead to highly imprecise rate estimates. This imprecision is amplified by MAUP, causing substantial variability in the identification of disease clusters based on different spatial units [4,36]. As a result, health interventions and resource allocations might be misdirected due to the unreliability of the spatial analysis.
>
> In addition, advantages of using the well-defined boundary levels of SA1 and SA2 also enables easy incorporation of additional demographic and socio-economic data into disease models [37]. Detailed spatial data at these levels can often be more effectively communicated to stakeholders, policymakers, and the public, facilitating better understanding and engagement.
>
> With higher aggregations, we found that the spatial autocorrelation of the data is no longer significant (for SA3 and SA4 levels), so use of spatial modelling/smoothing may not be required. But since SA1 and SA2 level data has significant spatial autocorrelation, analysis of cancer incidence data at these levels should be performed using a model that accounts for spatial autocorrelation.

References

1. Best N, Richardson S, Thompson A. A comparison of Bayesian spatial models for disease mapping. Statistical methods in medical research. 2005;14(1):35–59.

2. Openshaw S. The modifiable areal unit problem. Concepts and techniques in modern geography. 1984;.

3. Wong DW. The modifiable areal unit problem (MAUP). WorldMinds: geographical perspectives on 100 problems: commemorating the 100th anniversary of the association of American geographers 1904–2004. 2004; p. 571–575.

4. Fotheringham AS, Wong DW. The modifiable areal unit problem in multivariate statistical analysis. Environment and planning A. 1991;23(7):1025–1044.

5. Gregorio DI, DeChello LM, Samociuk H, Kulldorff M. Lumping or splitting: seeking the preferred areal unit for health geography studies. International Journal of Health Geographics. 2005;4:1–10.

6. Tuson M, Yap M, Kok MR, Murray K, Turlach B, Whyatt D. Incorporating geography into a new generalized theoretical and statistical framework addressing the modifiable areal unit problem. International journal of health geographics. 2019;18(1):1–15.

7. Buzzelli M. Modifiable areal unit problem. International encyclopedia of human geography. 2020; p. 169.

8. Nelson JK, Brewer CA. Evaluating data stability in aggregation structures across spatial scales: revisiting the modifiable areal unit problem. Cartography and Geographic Information Science. 2017;44(1):35–50.

9. Manley D. Scale, Aggregation, and the Modifiable Areal Unit Problem. In: Handbook of regional science. Springer; 2013. p. 1157–1171.

10. Marí-Dell'Olmo M, Oliveras L, Vergara-Hernández C, Artazcoz L, Borrell C, Gotsens M, et al. Geographical inequalities in energy poverty in a

Mediterranean city: Using small-area Bayesian spatial models. Energy Reports. 2022;8:1249–1259.

11. Kok MR, Tuson M, Yap M, Turlach B, Boruff B, Vickery A, et al. Impact of the modifiable areal unit problem in assessing determinants of emergency department demand. Emergency Medicine Australasia. 2021;33(5):794–802.

12. Dapena AD, Vázquez EF, Morollón FR. The role of spatial scale in regional convergence: the effect of MAUP in the estimation of β-convergence equations. The Annals of Regional Science. 2016;56(2):473–489.

13. Jeffery C, Ozonoff A, Pagano M. The effect of spatial aggregation on performance when mapping a risk of disease. International journal of health geographics. 2014;13(1):1–9.

14. Tuson M, Yap M, Kok MR, Boruff B, Murray K, Vickery A, et al. Overcoming inefficiencies arising due to the impact of the modifiable areal unit problem on single-aggregation disease maps. International journal of health geographics. 2020;19(1):1–18.

15. Besag J, York J, Mollié A. Bayesian image restoration, with two applications in spatial statistics. Annals of the Institute of Statistical Mathematics. 1991;43(1):1–20.

16. Fontanet CP, Carlos H, Weiss JE, Diaz MCG, Shi X, Onega T, et al. Evaluating Geographic Health Disparities in Cancer Care: Example of the Modifiable Areal Unit Problem. Annals of Surgical Oncology. 2023;30(12):6987–6989.

17. Shi X, Miller S, Mwenda K, Onda A, Rees J, Onega T, et al. Mapping disease at an approximated individual level using aggregate data: a case study of mapping New Hampshire birth defects. International Journal of Environmental Research and Public Health. 2013;10(9):4161–4174.

18. Cancer Council Queensland. Simulated Lung cancer data for Queensland; 2015. Email: Statistics@cancerqld.org.au.

19. ABS. Australian statistical geography standard (ASGS); 2016. Available from: https://www.abs.gov.au/AUSSTATS/abs@.nsf/allprimarymainfeatures/871A7FF33DF471FBCA257801000DCD5F?opendocument.

20. ABS. Main Features-Overview; 2016. Available from: https://www.abs.gov.au/ausstats/abs@.nsf/Lookup/by%20Subject/1270.0.55.001~July%202016~Main%20Features~Overview~1.

21. Wong MC, Lao XQ, Ho KF, Goggins WB, Tse SL. Incidence and mortality of lung cancer: global trends and association with socioeconomic status. Scientific reports. 2017;7(1):14300.

22. ABS. Socio-Economic Indexes for Areas; 2016. Available from: https://www.abs.gov.au/ausstats/abs@.nsf/mf/2033.0.55.001.

23. ABS. 2033.0.55.001 - Census of Population and Housing: Socio-Economic Indexes for Areas (SEIFA), Australia, 2016 — .nsf; 2016. https://www.abs.gov.au/AUSSTATS/abs@.nsf/DetailsPage/2033.0.55.0012016.

24. ABS. Australian Bureau of Statistics; 2016. Available from: https://www.abs.gov.au/.

25. Cliff A, Ord J. The Problem of spatial autocorrelation. Papers in Regional Science 1, Studies in Regional Science, 25-55, edited by A. J Scott; 1969.

26. Tiefelsdorf M. Modelling spatial processes: the identification and analysis of spatial relationships in regression residuals by means of Moran's I. Springer; 2000.

27. Kim D. Predicting the magnitude of residual spatial autocorrelation in geographical ecology. Ecography. 2021;44(7):1121–1130.

28. Sue Bell B, Broemeling LD. A Bayesian analysis for spatial processes with application to disease mapping. Statistics in Medicine. 2000;19(7):957–974.

29. CARLIN BP, Xia H. Assessing environmental justice using Bayesian hierarchical models: two case studies. Journal of Exposure Analysis & Environmental Epidemiology. 1999;9(1).

30. Escaramís G, Carrasco JL, Ascaso C. Detection of significant disease risks using a spatial conditional autoregressive model. Biometrics. 2008;64(4):1043–1053.

31. Lee D. CARBayes: an R package for Bayesian spatial modeling with conditional autoregressive priors. Journal of Statistical Software. 2013;55(13):1–24.

32. R Core Team. R: A Language and Environment for Statistical Computing; 2016. Available from: https://www.R-project.org/.

33. Lam P. Convergence diagnostics. Lecture presented at Government. 2002;.

34. Bivand R, Altman M, Anselin L, Assunção R, Berke O, Bernat A, et al.. Package 'spdep'; 2015. Available from: https://cran.r-project.org/web/packages/spdep/index.html.

35. ABS. Mesh Blocks; Jul 2021 - Jun 2026. Available from: https://www.abs.gov.au/statistics/standards/australian-statistical-geography-standard-asgs-edition-3/jul2021-jun2026/main-structure-and-greater-capital-city-statistical-areas/mesh-blocks.

36. Amrhein CG. Searching for the elusive aggregation effect: evidence from statistical simulations. Environment and planning A. 1995;27(1):105–119.

37. Kang SY, McGree J, Mengersen K. The choice of spatial scales and spatial smoothness priors for various spatial patterns. Spatial and Spatio-temporal Epidemiology. 2014;10:11–26.
