# Supplementary material for: Assessing the influence of the modifiable areal unit problem on Bayesian disease mapping in Queensland, Australia
Source: PLoS One. 2025 Jan 28;20(1):e0313079. doi: 10.1371/journal.pone.0313079 (PMC11774366; doi:10.1371/journal.pone.0313079)
Supplement: Attachment — Submitted filename: PONE-D-24-13684_Review Comments.docx [file pone.0313079.s002.docx]

1. Why is it significant to investigate the impact of the MAUP in regions with sparse data, particularly in countries like Australia with less populated areas?

2. How MAUP affects the analysis of spatially aggregated data? Why it is a concern in spatial analysis?

3. What advantages do area structures with moderate resolution offer in terms of dealing with the MAUP?

4. What types of Bayesian spatial models were employed in the study, and how do they account for spatial correlation and variability?

5. What are the potential benefits of using SA1 or SA2 levels for spatial modelling according to the authors?
